# Conical Nanotubes Synthesized by Atomic Layer Deposition of Al_2_O_3_, TiO_2_, and SiO_2_ in Etched Ion-Track Nanochannels

**DOI:** 10.3390/nano11081874

**Published:** 2021-07-21

**Authors:** Nils Ulrich, Anne Spende, Loïc Burr, Nicolas Sobel, Ina Schubert, Christian Hess, Christina Trautmann, Maria Eugenia Toimil-Molares

**Affiliations:** 1 Materialforschung, GSI Helmholtzzentrum für Schwerionenforschung, 64291 Darmstadt, Germany; anne.spende@gmail.com (A.S.); loicburr@gmail.com (L.B.); i.schubert@gsi.de (I.S.); c.trautmann@gsi.de (C.T.); 2 Material-und Geowissenschaften, Technische Universität Darmstadt, 64287 Darmstadt, Germany; 3 Eduard-Zintl-Institut für Anorganische und Physikalische Chemie, Technische Universität Darmstadt, 64287 Darmstadt, Germany; nicolas.sobel@gmail.com (N.S.); christian.hess@tu-darmstadt.de (C.H.)

**Keywords:** conical nanotube, ion-track technology, atomic layer deposition, nanopore confinement, etched ion-track membrane, polymeric nanochannel, ion-current rectification

## Abstract

Etched ion-track polycarbonate membranes with conical nanochannels of aspect ratios of ~3000 are coated with Al_2_O_3_, TiO_2_, and SiO_2_ thin films of thicknesses between 10 and 20 nm by atomic layer deposition (ALD). By combining ion-track technology and ALD, the fabrication of two kinds of functional structures with customized surfaces is presented: (i) arrays of free-standing conical nanotubes with controlled geometry and wall thickness, interesting for, e.g., drug delivery and surface wettability regulation, and (ii) single nanochannel membranes with inorganic surfaces and adjustable isoelectric points for nanofluidic applications.

## 1. Introduction

Surface coating by atomic layer deposition (ALD) has the potential to significantly widen the technological and scientific applications of etched ion-track membranes with highly oriented and monodisperse channels by enabling a controlled tailoring of both size and chemistry of the nanochannels. ALD is based on sequential and self-terminating gas–solid surface reactions of typically two gaseous reactants and has been successfully applied to coat complex three-dimensional topographies with homogeneous films of known composition [1,2,3,4,5]. The self-limiting nature of the chemical reactions provides excellent thickness control, even in deeply embedded surfaces such as the inner wall of nanopores and nanochannels. In recent years, conformal TiO_2_, SiO_2_, and Al_2_O_3_ coating of cylindrical nanochannels in porous alumina membranes [6,7] and in etched ion-track polycarbonate membranes with aspect ratios (length over diameter) up to 3000 [8,9,10,11,12,13,14,15,16] was demonstrated. In the latter case, specific low-temperature ALD processes ensured the thermal stability of the polymer material [14,15]. ALD even allows coatings consisting of several layers of different materials. For example, multichannel PET membranes were ALD-coated with Al_2_O_3_ and ZnO layers, obtaining sub-10 nm channels, which were used to confine gramicidin A inside the channels [17].

The advantage of etched ion-track membranes is the fact that size, geometry, and density of the channels can be adjusted independently. During swift heavy ion irradiation, each ion creates an individual track of a few nanometers in diameter and several tens of micrometers in length. The energy deposited along the ion trajectory leads to severe damage, including broken molecular bonds [18]. Given the focus and the high energy of the ion beam, the tracks are parallel oriented and penetrate completely through the polymer membrane. The track density can be varied from 1 per sample to about 10^10^ per cm^2^ or more [19,20,21,22,23,24,25,26]. In a suitable etching solution, the damaged track material is selectively dissolved, converting each ion track into an individual open channel, whose shape is tailored by the etching conditions. When the etching solution attacks the ion tracks from both sides of the membrane, cylindrical nanochannels are synthesized. Asymmetric etching conditions lead to conical or bullet-like nanochannels [27,28,29,30,31]. In both cases, the nanochannel diameter increases with etching time and can be adjusted in a controlled manner between a few tens of nanometers and a few micrometers. The etching rate determines the growth of the channel diameter over etching time. It depends on various parameters, including composition and concentration of the etchant, temperature, and polymer type.

The most common ion-track membranes with symmetrically etched cylindrical channels are used for filtration and biotechnological applications and as templates for the synthesis of nanowires by electrodeposition [26,31]. Asymmetrically etched membranes with conically shaped channels have been applied as templates to fabricate free-standing conical nanowire arrays [32,33].

In addition, membranes with single nanochannels are being widely investigated regarding their ionic transport properties [34,35,36,37,38,39,40,41,42,43]. The ionic or molecular flow through a confined nanopore is affected by the channel geometry (length, diameter, shape) and surface chemistry. Solid-state nanochannels of sufficiently small diameter and charged surfaces commonly show ion current rectification [27,35,44,45], a characteristic that plays an important role in biological protein channels [46,47]. A broad range of approaches has been applied for surface modification and functionalization of asymmetric etched ion-track channels, including electroless deposition [48], chemical methods [36,49,50,51], and more recently ALD [52]. This has resulted in an enormous palette of nanochannel devices for nanofluidic applications [53,54,55] as well as for various chemical and biological sensors, e.g., for urea [43] and glucose sensors [56]. The transport and sensing properties of conical nanochannels are strongly influenced by their geometric parameters [57], and the characterization of the pore replica offers a valuable tool to measure the dimensions and morphology of a given nanopore [58].

In this work, polycarbonate multichannel membranes with conical nanochannels are uniformly coated by ALD. By removing the membrane material, we demonstrate the fabrication of arrays of SiO_2_, Al_2_O_3_, and TiO_2_ conical nanotubes with wall thicknesses between ~10 and ~20 nm. The mechanical stability of free-standing nanocones depends on both the material and thickness of the deposited wall. 

In addition, the ion transport properties of single conical ALD-coated nanochannels are investigated. By the coating process, the diameter of the pore tip can be reduced in a controlled manner and by selecting a specific coating material, the surface chemistry and charge of the channel wall can be tailored. ALD coating of asymmetric nanochannels with oxides is of interest because it provides flexibility in further functionalization steps, enlarging the opportunities of surface chemistry for novel nanochannel-based sensing devices.

## 2. Materials and Methods

**Figure 1:**
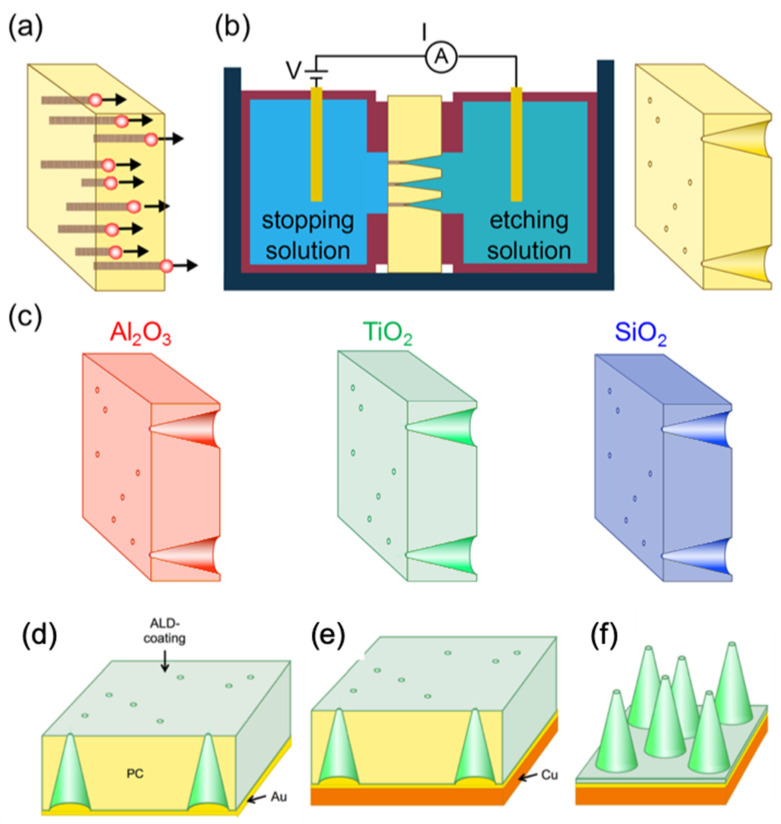
Fabrication of nanocone arrays by combining ion-track technology and ALD. (**a**) Swift heavy ion irradiation of polycarbonate foils, leading to parallel-oriented tracks. (**b**) Scheme of the electrochemical cell employed for asymmetric etching of the irradiated foils. Each ion track is converted into a conical nanochannel. (**c**) Conformal surface coating of etched membranes by low-temperature atomic layer deposition of Al_2_O_3_ (red), TiO_2_ (green), and SiO_2_ (blue). (**d**–**f**) Fabrication steps for free-standing tubular nanocone samples. (**d**) Sputtering of Au onto the coated membrane side with large nanochannel openings. (**e**) Reinforcing the sputtered Au layer by electrodeposition of additional Cu layer. (**f**) Free-standing tubular nanocones after dissolution of the polymer template.

Figure 1 shows the synthesis strategy adopted for the fabrication of ALD-coated membranes with asymmetric channels (a–c) and free-standing tubular nanocones (d–f). First, 30 µm thick polycarbonate foils (Makrofol N, Bayer, Leverkusen, Germany) of 3 cm in diameter are irradiated with ~2 GeV Au ions at the UNILAC linear accelerator of the GSI Helmholtz Centre for Heavy Ion Research, Darmstadt, Germany. The irradiation of the foils is performed under normal beam incidence with a fluence of 10^6^ ions/cm^2^ or alternatively with one single ion per sample. The irradiated polymer foils are then exposed to UV light for 1 h on each side and subsequently mounted between two compartments of an electrochemical cell. For asymmetric etching, one compartment is filled with a 60:40 mixture of 9 mol/L NaOH and CH_3_OH as etchant, while the other cell compartment contains deionized H_2_O (Millipore Direct-QTMS, Merck, Darmstadt, Germany) as a stopping solution. The cell is thermostated at a temperature of 30 °C. For track etching, a potential of +1 V is applied across the membrane. The etchant preferentially dissolves the ion tracks and converts the track damage into an open channel. Under the applied asymmetric etching conditions, asymmetric channels of conical geometry are obtained, exhibiting a large base and a small tip (b). Depending on the intended channel diameter, the etching process is stopped by removing the membrane from the cell and rinsing it with deionized water. To produce membranes with single conical nanochannels for ionic conductivity measurements, an etching time of ~15 min is chosen, leading to a base diameter of ~3 µm. In a next step, the polycarbonate membranes containing single or multiple conical nanochannels are coated by ALD with three different materials: Al_2_O_3_, TiO_2_, and SiO_2_. The ALD process for Al_2_O_3_ is applied at 110 °C and uses trimethylaluminium (25 °C, SAFC Hitech, Steinheim, Germany) as well as deionized water (25 °C) as precursors. For the TiO_2_ deposition, taking place at 110 °C [15], titanium isopropoxide (70 °C, SAFC Hitech, Steinheim, Germany) and deionized H_2_O (25 °C) are used as precursors. SiO_2_ is deposited at 60 °C [14], by application of silicon tetrachloride (25 °C, Sigma-Aldrich, Steinheim, Germany) and deionized water (25 °C) as precursors. The SiO_2_ coating is conducted by pyridine (C_5_H_5_N, anhydrous, Sigma-Aldrich, Steinheim, Germany) insertion into the reaction chamber in both reaction steps just before the precursor. Pyridine acts as a catalyst and limits the reaction temperature to 60 °C [14,59]. ALD layers of the three oxides deposited under such low temperatures exhibit an amorphous structure [60,61,62]. The growth rate for these low-temperature ALD processes is determined by ellipsometry of reference coatings on silicon wafers as well as by SEM analysis of the coated nanochannels, yielding 0.86 Å/cycle for Al_2_O_3_, 0.08 Å/cycle for TiO_2_, and 1.7 Å/cycle for SiO_2_. The number of ALD cycles is systematically varied to fabricate coatings with thicknesses between 10 and 20 nm.

Before and after ALD coating, the ionic conductivity of the single-channel membranes is investigated by inserting the membrane between the two chambers of an electrochemical cell and recording current–voltage (I–V) curves. By using two Au electrodes and scanning the voltage between ±1 V across the membrane, the current is monitored with a picoammeter/voltage sourcemeter (Keithley 6487, Solon, OH, USA). Ion conductivity measurements at room temperature are performed using a 1 M KCl solution (pH 5) that is pH adjusted by adding HCl (37%, Carl Roth GmbH, Karlsruhe, Germany) for measurements with pH 2 and disodium (hydrogen) phosphate (Na_2_HPO_4_, >99%, Sigma-Aldrich, Steinheim, Germany) for pH 9 as an electrolyte.

Thickness, homogeneity, and conformity of the ALD coating inside the conical nanochannels are visualized by dissolving the polycarbonate template in dichloromethane (>99.5%, Carl Roth GmbH, Karlsruhe, Germany) and collecting the released conical nanotubes on a standard Cu-lacey transmission electron microscopy grid (TEM grid). The nanotubes are characterized with a transmission electron detector (STEM-in-SEM) in a high-resolution scanning electron microscope (JEOL JSM-7401F, Akishima, Japan). The elemental composition is investigated by energy dispersive X-ray spectroscopy (EDX) using a Bruker Xflash 5030 EDX Spectrometer, Billerica, MA, USA.

To fabricate arrays of free-standing conical nanotubes (Figure 1d–f), the ALD-coated templates are mechanically stabilized by a metal layer on the base side before dissolving the polycarbonate. After the ALD process, a ~200 nm thin Au layer is sputtered onto the base side of the membrane using an Edwards Sputter Coater S150B (Figure 1d). To reinforce the Au layer as a substrate, Cu is electrodeposited using a copper sulfate based electrolyte (238 g/L Cu_2_SO_4_ and 21 g/L H_2_SO_4_) in a two-electrode configuration applying *U* = −0.5 V (vs. Cu rod) in an electrochemical cell. After 10 min of electrodeposition at room temperature, the Cu layer is about 10 µm thick (Figure 1e). Once the substrate is fabricated, the polymer foil is dissolved in dichloromethane. During the dissolution process, the thin planar ALD oxide layer on the top surface of the membrane is lifted off. The resulting nanocone arrays are visualized by SEM under 20° tilting. The nanotube replica of the single-nanochannel membranes for SEM visualization are prepared after the I–V conductivity measurements in the same way, i.e., sputtering a gold layer, reinforcement by copper deposition, and finally polymer dissolution. 

## 3. Results and Discussion

### 3.1. Transport Properties of ALD-Coated Single Conical Nanochannels

**Figure 2:**
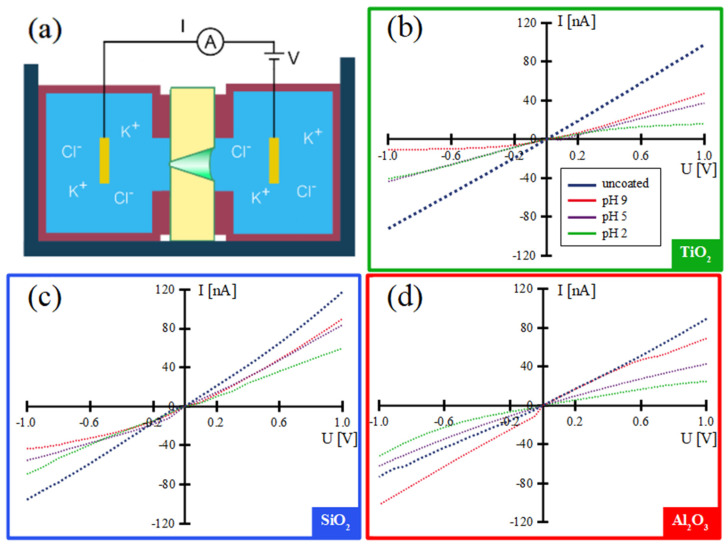
Scheme of electrochemical cell for I–V measurements (**a**). I–V curves for single conical nanochannels uncoated (blue) and coated with ~15 nm TiO_2_ (**b**), SiO_2_ (**c**), and Al_2_O_3_ (**d**) at different pH values. I–V curves were recorded in a 1 M KCl solution adjusted to pH values of 2 (green), 5 (purple), and 9 (red).

The ALD processes for Al_2_O_3_, TiO_2_, and SiO_2_ are applied to coat membranes containing one single conical nanochannel. The ionic conductivity of these channels is measured before and after ALD coating in an electrochemical cell (Figure 2a) by recording I–V curves between ±1 V across the membrane (Figure 2b–d). The conductivity of the single channel is given by

(1) $$\frac{I}{U}=\frac{\pi *\kappa *d*D}{4*L}$$

with I being the recorded electrical current, U the voltage applied between the two Au electrodes, κ the electrical conductivity of the electrolyte, L the length of the nanochannel, d its tip diameter, and D the base diameter [35].

The uncoated membrane is measured at pH 5, close to the isoelectric point (IEP) of polycarbonate. At this value, the polymer surface is not charged [63], and the I–V curve is rather linear, indicating symmetric ion transport along the nanochannel.

In a first run, I–V measurements on the ALD-coated single channels are performed at a specific pH value, which is close to the isoelectric point of the different materials, i.e., pH 9 for Al_2_O_3_, pH 5 for TiO_2_, and pH 2 for SiO_2_ [64]. At pH ~IEP, the nanochannel surface is uncharged and both positive and negative ions flow unhindered through the nanochannel [65]. Consistently, the I–V curves obtained at pH 5 for TiO_2_-coated and pH 2 for SiO_2_-coated nanochannels are linear, i.e., they show no rectification behavior (Figure 2b,c). In contrast, the I–V curve for the Al_2_O_3_-coated conical nanochannel at pH 9 (Figure 2d) exhibits a non-linear behavior with a small rectification factor of *f_rec_* = 1.5 (*f_rec_* = (*I* (−1 V)/*I* (+1 V). This slight deviation is possibly due to the poor stability of Al_2_O_3_ in aqueous solutions, which may lead to local surface charge inhomogeneities [66]. From these I–V curves measured at pH values at the respective IEPs of the surface material, we deduce the diameter of the nanochannel tip before and after the different ALD coatings. The tip diameter d is calculated by Equation (2), which is the transformed version of the conductivity formula (Equation (1)).

(2) $$d=\frac{4*L*I}{U*D*\pi *\kappa }$$

The nanochannel length L corresponds to the thickness of the PC membrane, the pore base diameter D is deduced from SEM images of the corresponding free-standing single nanocone replica (see section below), and the specific conductivity κ is 10.68 Sm^−1^ at 25 °C for a 1 M KCl solution [67]. Table 1 presents the tip and base diameters of the single nanopores before and after the ALD coating process.

For the single nanochannel coated with TiO_2_, a tip diameter of ~107 nm before and ~71 nm after ALD coating is calculated. For the SiO_2_-coated nanochannel, the tip diameter was ~106 nm before and ~72 nm after ALD coating. For the Al_2_O_3_-coated nanochannel, the respective tip diameters were ~103 nm before and ~74 nm after the ALD coating. In all three cases, the measured difference in tip diameter before and after coating is in excellent agreement with the nominal ALD coating thickness of 15 nm.

Subsequently, the ionic conductivity of each coated nanopore is measured at two other pH values, one higher and one lower than the IEP. Depending on the pH value, the surface charge of the nanochannel wall changes. This has a direct influence on the electrical double layer (EDL) and thus on the ion flow along the nanochannel, especially at the tip where the diameter is small. For the TiO_2_-coated nanochannel (Figure 2b), ion current rectification factors of *f_rec_* = 2.6 at pH = 2, *f_rec_* = 1.2 at pH = 5, and *f_rec_* = −4.4 at pH = 9 are obtained. The negative surface charge is expressed by a negative rectification factor [68]. The TiO_2_-coated nanopore shows no rectification at a solution pH = 5 (equal to the surface IEP), as anticipated. However, for a smaller pH value, a positive rectification factor and, for a higher pH value, a negative rectification factor are obtained, indicating that a TiO_2_-coated conical nanopore can act as a cation or anion selector depending on the pH value of the environment.

For the SiO_2_-coated nanopore (Figure 2c), the IEP is ~2. Higher pH values result in a rectification factor of *f_rec_* = 1.5 at pH = 5 and *f_rec_* = −2.1 at pH = 9. Obviously, the more negatively the surface is charged, the more anions are hindered in passing through the nanochannel. This observation is in agreement with the Derjaguin-Landau-Verwey-Overbeek theory [65].

For the Al_2_O_3_-coated nanochannel, the variation of the rectification factor *f_rec_* as a function of the pH value is almost negligible, being *f_rec_ =* 1.5 at pH = 9, *f_rec_ =* 1.5 at pH = 5, and *f_rec_ =* 2.1 at pH = 2 (Figure 2d). The rectification factor is positive in all cases, because at pH values lower than the IEP, the channel surface is charged positively. We also attribute the weak pH dependency to the instability of the Al_2_O_3_ layer in aqueous solution, although this remains to be clarified.

These results clearly show that by ALD coating with different oxides, the IEP of a single conical nanopore can be adjusted, resulting in the creation of nanopores with anion, cation, or ambivalent selectivity.

The determined rectification factors are low due to the fact that the tip diameters of our nanochannels are rather large (between ~70 and 100 nm) compared to the EDL thickness (~0.3 nm for 1 M KCl electrolyte [65]). Ion current rectification in relatively large channels has been reported previously [69,70] and numerical simulations explain this phenomenon by a concentration of the nanopore surface charge at the tip, resulting in a local increase in the EDL thickness [71].

Finally, it also should be mentioned that ALD-coated single conical nanochannels exhibited much higher stability than uncoated nanochannels: I–V measurements could be performed for three months compared to several days for uncoated channels. We therefore consider ALD coating as a promising process for nanochannel applications that require good long-term stability.

**Figure 3:**
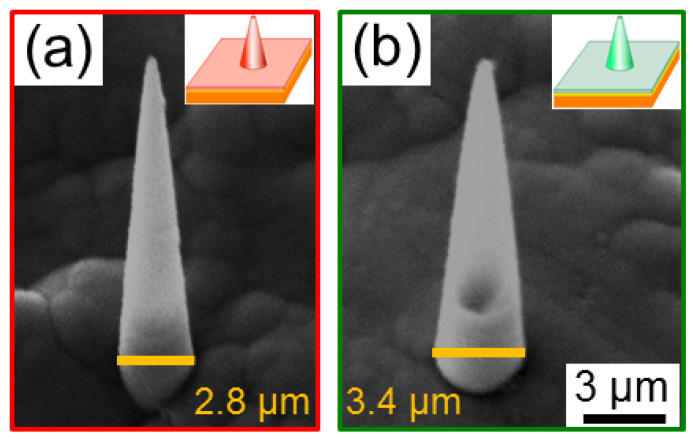
SEM images of free-standing single conical nanotubes of Al_2_O_3_ (**a**) and TiO_2_ (**b**). The cones are stable although the tube wall consists of a ~15 nm thick ALD layer.

In order to gain additional access to the geometrical parameters, first a stabilizing Au layer is sputtered onto the bottom side and is reinforced by an electrodeposited Cu layer, which protrudes a few µm into the nanotubes. The nanocones are then released from the membranes by dissolving the polymer. SEM images of single free-standing conical Al_2_O_3_ and TiO_2_ nanotubes are shown in Figure 3a,b. The cones consist of a ~15 nm thick ALD layer. Since the tip diameter is too small to be determined from SEM images, we measure the base diameter D and insert this value into Equation (2) together with the data obtained from the I–V measurements for the tip diameter calculation. The base diameters determined for the single cone membranes are in good agreement with reference samples synthesized in multichannel membranes under the same etching conditions.

### 3.2. Geometrical and Compositional Characterization 

**Figure 4:**
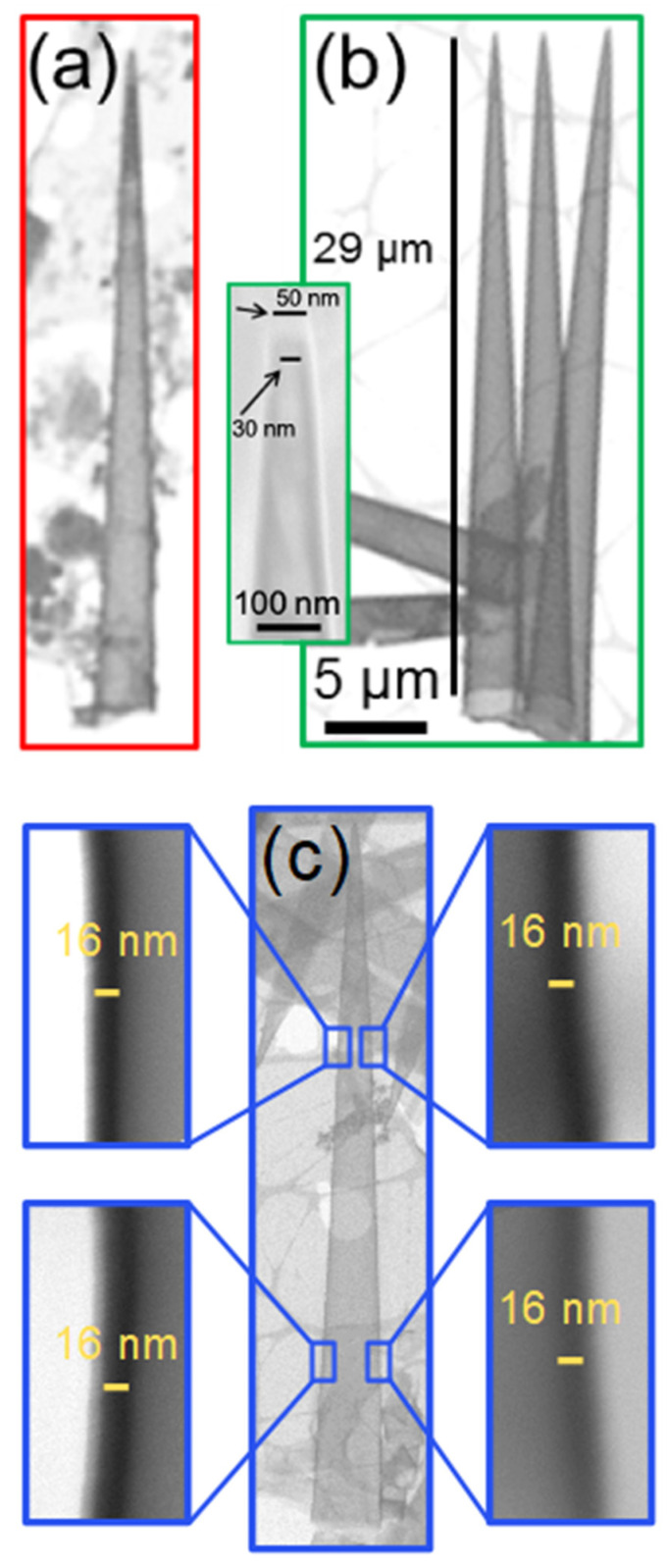
STEM-in-SEM images of ~30 µm long conical Al_2_O_3_ (**a**), TiO_2_ (**b**), and SiO_2_ (**c**) nanotubes with a wall thickness of ~16 nm. The inset in (**b**) shows the open tip of a TiO_2_ tubular nanocone (~16 nm wall thickness) in detail. Images with higher magnification in (**c**) demonstrate a homogeneous wall thickness along the entire cone length. The scale bar shown in (**b**) also applies for (**a**–**c**).

In the following section, we further characterize the geometry and composition of ALD-fabricated nanotube arrays released from multichannel templates. Figure 4 shows STEM-in-SEM images of representative tubular cones of (a) Al_2_O_3_, (b) TiO_2_, and (c) SiO_2_ exhibiting a nominal wall thickness of ~16 nm. For all three materials, ALD coating produces a homogeneous shape-conforming replica of the track-etched conical nanopores along their entire length of 30 µm. The high magnification image in Figure 4c gives evidence that the SiO_2_ wall has a thickness of ~16 nm along the full length of the nanocone. The smoothness of the wall is in agreement with earlier investigations on ALD-coated cylindrical channels in polycarbonate [15] and confirms the smooth channel walls reported for PC in contrast to the rather rough walls of track-etched polyethylene terephthalate (PET) membranes [69,72]. The inset of Figure 4b shows the tip of a TiO_2_ nanocone. The inner and outer diameters of the tip are approximately 30 nm and 50 nm, respectively. Due to the small size, SEM focusing of the tip is challenging and we are faced with the uncertainty that the original top of the tip may have been broken off.

**Figure 5:**
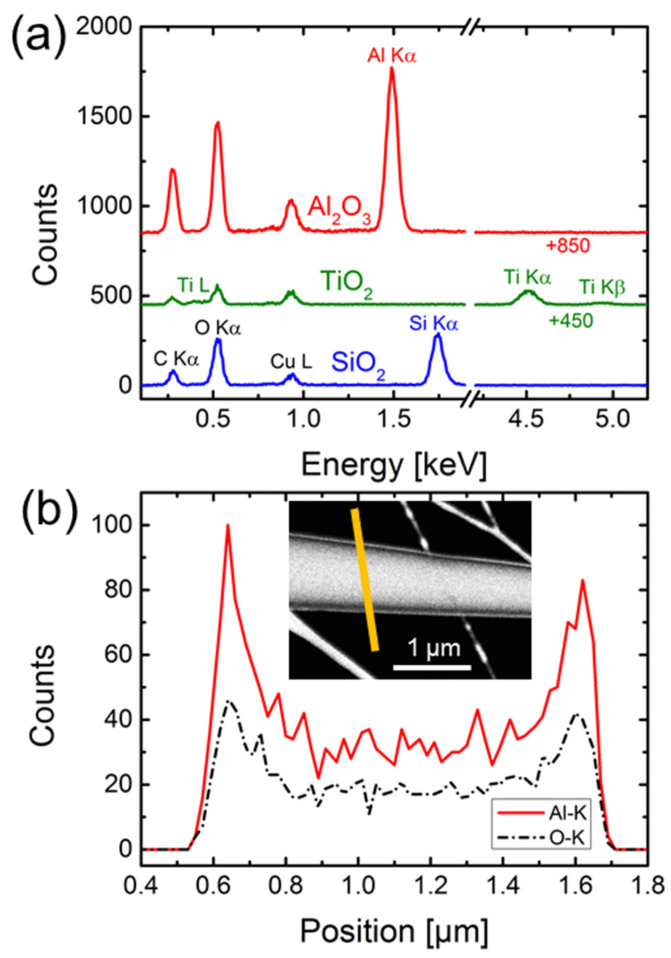
(**a**) EDX multipoint spectra of Al_2_O_3_, TiO_2_, and SiO_2_ nanocones with a wall thickness of ~20 nm, lying on a lacey Cu-TEM grid. (**b**) EDX linescan across an Al_2_O_3_ nanocone. In the STEM dark field image, the position of the linescan is marked in yellow. The intensity distribution of the corresponding aluminum (red solid line) and oxygen (black dashed line) signals confirms the tubular shape of the nanocones.

Figure 5a shows EDX multipoint spectra of Al_2_O_3_, TiO_2_, and SiO_2_ conical nanotubes with a wall thickness of ~20 nm. The spectra present the corresponding elemental peaks of aluminum, titanium, silicon, and oxygen. Copper and carbon peaks are contributions from the Cu-lacey TEM grids. The height of the carbon signal varies, depending on the position of the nanocone with respect to the carbon support film. These EDX spectra clearly demonstrate that the ALD process within the conical polycarbonate nanochannels leads to pure and well-defined coating layers for all three materials. In addition, EDX linescans are taken to display the material distribution in the nanocones. Figure 5b displays the EDX linescan across an Al_2_O_3_ nanocone and the corresponding STEM-in-SEM image (inset) with the position of the recorded EDX marked in yellow. The intensity of the aluminum and oxygen signals is high at the position of the tube walls and drops between the walls, which gives further evidence of the tubular shape of the nanocones. 

### 3.3. Free-Standing Conical Nanotube Arrays

Arrays of high-aspect-ratio conical nanotubes are successfully fabricated by mechanically stabilizing the ALD-coated multichannel membranes prior to the dissolution of the polymer membrane (cf. Figure 1d–f). The intimate contact between the metal supporting layer and the micrometer-sized bases of the ALD-coated channels stabilizes the sample during its immersion in dichloromethane for dissolution of the polymer and avoids the dispersion of the conical tubes in the solution. 

**Figure 6:**
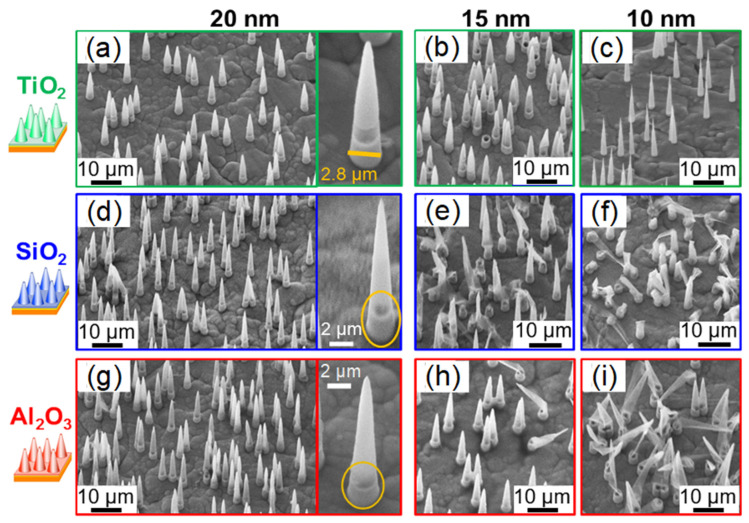
SEM images of TiO_2_ (**a**–**c**), SiO_2_ (**d**–**f**), and Al_2_O_3_ (**g**–**i**) free-standing nanocones with wall thicknesses of ~20 nm, ~15 nm, and ~10 nm. The sputtered Au and electrodeposited Cu close to the cone base are visible at a higher magnification (single cones in **a**,**d**,**g**). The images are recorded under a tilting angle of 20°.

Figure 6 shows representative SEM images of the resulting TiO_2_ (a–c), SiO_2_ (d–f), and Al_2_O_3_ (g–i) nanocone arrays with wall thicknesses of ~20 nm (left), ~15 nm (center), and ~10 nm (right). For all samples, the number of cones per cm^2^ counted at various positions is in excellent agreement with the number of channels in the membrane, demonstrating that all nanochannels were coated. The wall thickness is determined from reference samples coated simultaneously and analyzed by STEM-in-SEM (see Figure 4). We find that the thickness for all analyzed conical tubes is the same, evidencing that the ALD process was homogeneous across the whole membrane area, as expected. 

After the release from the polycarbonate template, all cones with a wall thickness of ~20 nm are free-standing and stable (Figure 6a,d,g). For a wall thickness of ~15 nm, many SiO_2_ and some Al_2_O_3_ cones are broken (Figure 6b,e,h). This instability is even more pronounced if the wall is only ~10 nm thick (Figure 6c,f,i). The damage is partially ascribed to mechanical forces during the dissolution process of the template and sample handling. However, we also observe bending and finally collapsing of Al_2_O_3_ and SiO_2_ nanocones in situ when the electron beam is kept focused on a nanocone for longer periods of time. Thin Al_2_O_3_ conical tubes are most severely affected by charging effects under the SEM electron beam. It is striking that almost none of the TiO_2_ cones are broken (Figure 6a–c), including the very thin ones with a ~10 nm wall thickness (Figure 6c). The fracture toughness values for bulk material are ~0.77 MPa m^1/2^ for SiO_2_ [73], ~2.8 MPa m^1/2^ for TiO_2_ [74], and ~5 MPa m^1/2^ for Al_2_O_3_ [75]. The low fracture toughness may explain the instability of the SiO_2_ tubes. In contrast, the remarkable stability of the TiO_2_ conical nanotubes can be attributed to size effects. In TiO_2_ low-dimensional systems, it was reported that refinement of the TiO_2_ grains leads to crack arrest and fracture toughness values up to 27 MPa m^1/2^ [76]. This effect may also stabilize the thin ALD walls of our TiO_2_ nanocones.

## 4. Conclusions

Al_2_O_3_, SiO_2_, and TiO_2_ conical nanotubes with wall thicknesses between 10 and 20 nm are fabricated by ALD coating of polycarbonate multi- and single-ion-track etched membranes with conical nanochannels. By dissolving the polycarbonate template, arrays of free-standing nanotubes are obtained. The released cones are homogeneous and intact, demonstrating that the ALD process yields conformal coating along the whole 30 µm of the conical nanochannels. The mechanical stability of nanocones with a wall thickness between 15 and 20 nm is relatively high, whereas nanocones with 10 nm thick walls become slightly fragile. Those free-standing conical nanotube arrays can be potentially applied as platforms for the investigation of drug delivery to cells and cell–nanostructure interactions [8]. Due to their stability, the conical nanotubes also provide an excellent replica for studying the geometrical structure of synthetic nanopores.

The coating of single-channel membranes with Al_2_O_3_, SiO_2_, and TiO_2_ changes the isoelectric point of the surface and leads to preferential cation, anion, and ambivalent selectivity of ionic transport. Thus, ALD-coated conical nanopores offer excellent systems for nanofluidic studies. The possibility to regulate ionic transport in a controlled manner offers a potential application for nanometer-sized electric devices, e.g., nano field effect transistors.

The ALD technology in combination with track-etched membranes provides interesting hybrid systems and creates new possibilities for tailored ion transport systems. ALD coating of the polymer template produces an inorganic surface with a defined IEP. The coating maintains the flexible character of the membrane, increases the long-term stability of the system, and offers new perspectives for tailoring nanofluidic systems, where ALD coatings provide a novel base for surface functionalization.

## Figures and Tables

**Table 1:** Tip and base diameter of the single conical nanopores.

Coating Material	Tip Diameter Uncoated	Tip Diameter Coated
TiO_2_	107 nm	71 nm
SiO_2_	106 nm	72 nm
Al_2_O_3_	103 nm	74 nm

## Data Availability

The data presented in this study are available on request from the corresponding author.
